# Azithromycin drives alternative macrophage activation and improves recovery and tissue sparing in contusion spinal cord injury

**DOI:** 10.1186/s12974-015-0440-3

**Published:** 2015-11-24

**Authors:** Bei Zhang, William M. Bailey, Timothy J. Kopper, Michael B. Orr, David J. Feola, John C. Gensel

**Affiliations:** Spinal Cord and Brain Injury Research Center, Department of Physiology, University of Kentucky, Lexington, KY 40536 USA; Integrated Biological Sciences Graduate Program, College of Medicine, University of Kentucky, Lexington, KY 40536 USA; Department of Pharmacy Practice and Science, College of Pharmacy, University of Kentucky, Lexington, KY 40536 USA; Spinal Cord and Brain Injury Research Center, University of Kentucky, B463 Biomed & Biological Science Research Building (BBSRB), 741 S. Limestone Street, Lexington, KY 40536-0509 USA

**Keywords:** Macrolide antibiotic, Spinal cord injury, Alternatively activated, Macrophage, Microglia, Azithromycin, M1, M2

## Abstract

**Background:**

Macrophages persist indefinitely at sites of spinal cord injury (SCI) and contribute to both pathological and reparative processes. While the alternative, anti-inflammatory (M2) phenotype is believed to promote cell protection, regeneration, and plasticity, pro-inflammatory (M1) macrophages persist after SCI and contribute to protracted cell and tissue loss. Thus, identifying non-invasive, clinically viable, pharmacological therapies for altering macrophage phenotype is a challenging, yet promising, approach for treating SCI. Azithromycin (AZM), a commonly used macrolide antibiotic, drives anti-inflammatory macrophage activation in rodent models of inflammation and in humans with cystic fibrosis.

**Methods:**

We hypothesized that AZM treatment can alter the macrophage response to SCI and reduce progressive tissue pathology. To test this hypothesis, mice (C57BL/6J, 3-month-old) received daily doses of AZM (160 mg/kg) or vehicle treatment via oral gavage for 3 days prior and up to 7 days after a moderate-severe thoracic contusion SCI (75-kdyn force injury). Fluorescent-activated cell sorting was used in combination with real-time PCR (rtPCR) to evaluate the disposition and activation status of microglia, monocytes, and neutrophils, as well as macrophage phenotype in response to AZM treatment. An open-field locomotor rating scale (Basso Mouse Scale) and gridwalk task were used to determine the effects of AZM treatment on SCI recovery. Bone marrow-derived macrophages (BMDMs) were used to determine the effect of AZM treatment on macrophage phenotype in vitro.

**Results:**

In accordance with our hypothesis, SCI mice exhibited significantly increased anti-inflammatory and decreased pro-inflammatory macrophage activation in response to AZM treatment. In addition, AZM treatment led to improved tissue sparing and recovery of gross and coordinated locomotor function. Furthermore, AZM treatment altered macrophage phenotype in vitro and lowered the neurotoxic potential of pro-inflammatory, M1 macrophages.

**Conclusions:**

Taken together, these data suggest that pharmacologically intervening with AZM can alter SCI macrophage polarization toward a beneficial phenotype that, in turn, may potentially limit secondary injury processes. Given that pro-inflammatory macrophage activation is a hallmark of many neurological pathologies and that AZM is non-invasive and clinically viable, these data highlight a novel approach for treating SCI and other maladaptive neuroinflammatory conditions.

**Electronic supplementary material:**

The online version of this article (doi:10.1186/s12974-015-0440-3) contains supplementary material, which is available to authorized users.

## Background

Spinal cord injury (SCI) triggers a CNS macrophage response consisting of pro-inflammatory, classically activated cells and anti-inflammatory, alternatively activated cells [1, 2]. Pro-inflammatory macrophages are neurotoxic, while anti-inflammatory macrophages promote axon growth and remyelination without concurrent neurotoxicity. Unfortunately, macrophages are polarized toward a pro-inflammatory phenotype after human and rodent SCI [1–4], and it is believed that these cells contribute to secondary injury processes. Indeed, TNF-α, iron, or age-related shifts toward pro-inflammatory macrophages are detrimental for SCI recovery [5–7]. In contrast, increasing anti-inflammatory macrophage activation through transplantation, adoptive transfer, or selective monocyte recruitment improves functional recovery [8–10]. These approaches illustrate the therapeutic potential of altering macrophage phenotypes on SCI recovery and repair; however, as a field, we are challenged to identify non-invasive, clinically viable, pharmacological techniques for altering SCI macrophage activation.

Macrophages are plastic and can adopt dynamic phenotypic and functional properties in response to new stimuli [11]. The pro-inflammatory SCI environment potentiates a pathological macrophage phenotype [3, 6]. However, through pharmacological interventions, it is possible to alter the way macrophages respond to pro-inflammatory stimuli. Azithromycin (AZM) is a macrolide antibiotic commonly used to treat infections in SCI individuals [12, 13]. In addition to its antibiotic properties, AZM increases alternative macrophage activation in rodent models of lung infection, skin inflammation, and sepsis; in alveolar macrophage and human monocyte cultures when incubated with pro-inflammatory stimulants; and in humans with cystic fibrosis [14–21]. Specifically, we previously observed that macrophages activated with pro-inflammatory stimuli adopt an anti-inflammatory phenotype in the presence of AZM [14]. Despite its reported immunomodulatory effects and safe pharmacological properties, the neurotherapeutic potential of altering macrophage activation with AZM in CNS disorders and trauma has not been examined.

In the present study, we used AZM to increase pro-reparative, alternative macrophage activation in the injured mouse spinal cord. In vivo, AZM significantly increased gene expression indicative of anti-inflammatory macrophage activation and reduced macrophage pro-inflammation gene expression. AZM treatment resulted in significantly increased functional recovery and less long-term tissue damage. In vitro, AZM drove anti-inflammatory cytokine production in response pro-inflammatory stimuli and rendered pro-inflammatory macrophages non-toxic. Collectively, these data illustrate the therapeutic potential of pharmacologically manipulating macrophages in SCI and identify AZM as a novel tool and therapeutic for application in neuroinflammatory conditions.

## Methods

### Experimental design

Mice were treated with AZM (160 mg/kg/day) or vehicle for 3 days prior to a moderate-severe contusion SCI. Drug administration was continued daily up to 7 days post injury (dpi). At 1, 3, and 7 dpi, *n* = 3–5 animals/treatment group were sacrificed and spinal cord tissue harvested for cell phenotypic analysis using fluorescent-activated cell sorting (FACS). Cells sorted from 3 and 7 dpi were further phenotypically evaluated using quantitative real-time PCR (rtPCR). Locomotor analyses (Basso Mouse Scale (BMS) and gridwalk) were conducted on a separate set of animals (*n* = 8–10/group) over the course of 4 weeks. At 28 dpi, these animals were sacrificed and spinal cord sections generated for histological analyses of tissue sparing and macrophage phenotype. In vitro studies were conducted using bone marrow-derived macrophages (BMDM) from adult mice. BMDMs were stimulated with AZM and/or pro-inflammatory stimuli (LPS + interferon-gamma (INFγ)). Control cells were left unstimulated. Secreted interleukin (IL)-10 and IL-12 levels were determined in the BMDM supernatant through ELISA analysis. The neurotoxicity of the BMDM supernatants was determined using the MTT assay to quantify the viability of supernatant-treated Neuro-2a cells.

### Animals

Experiments were performed using 4-month-old female C57BL/6 mice (Jackson Laboratory, Bar Harbor, Maine). Animals were housed in IVC cages with ad libitum access to food and water. All procedures were performed in accordance with the guidelines and protocols of the Office of Research Integrity and with approval of the Institutional Animal Care and Use Committee at the University of Kentucky.

### Spinal cord injury

Animals were anesthetized via intraperitoneal (i.p.) injections of ketamine (100 mg/kg) and xylazine (10 mg/kg). Following a T9 laminectomy, a moderate-severe thoracic SCI was produced using the Infinite Horizon (IH) injury device (75-kdyn displacement; Precision Systems and Instrumentation) [22]. Any animals receiving SCI with abnormalities in the force vs. time curve generated by the IH device were excluded from analysis. These abnormalities are indicative of bone hits or instability in the spinal cord at the time of injury and occurred <10 % of the time. Mice receiving a laminectomy without injury were used as sham controls. After injury, muscle and skin incisions were closed using monofilament suture. Post surgically, animals received one subcutaneous injection of buprenorphine-SR (1 mg/kg) and antibiotic (5 mg/kg, enroloxacin 2.27 %: Norbrook Inc., Lenexa, KS) in 2 ml of saline and were housed in warming cages overnight. Animals continued to receive antibiotic subcutaneously in 1 ml saline for 5 days. Azithromycin (160 mg/kg) or vehicle (1 % methylcellulose) was delivered in 0.1-ml volume via oral gavage daily beginning 3 days prior and continuing for 7 days post injury. Food and water intake and the incision site were monitored throughout the course of the study. Bladder expression was performed on injured mice twice daily.

### Cell isolation and phenotyping for flow cytometry

Following i.p. injection of ketamine (120 mg/kg) and xylazine (10 mg/kg), mice were transcardially perfused with ice cold diethyl pyrocarbonate phosphate-buffered saline (DEPC-PBS) then 1 cm of spinal cord centered on the injury site was rapidly dissected and placed in ice cold DEPC-PBS. The tissue was dissociated on ice using a size 40 mesh cell dissociation kit (Sigma, S0770) and rinsed twice with PBS. The dissociated tissue was then passed through a 70-μm screen filter (BD:352350). Cells were centrifuged at 200×*g* for 10 min at 4 °C, resuspended in fetal bovine serum (FBS) staining buffer (BD: 554656), and then cell numbers for each animal acquired using a hemocytometer. Cells were incubated with Fc block (BD:553142) for 15 min on ice and then were incubated with CD11b-APC, GR1-PE-Cy7, CD45-PerCP-Cy5.5, and CD206 (mannose receptor)-PE antibodies (BD Biosciences) as previously described [17]. Cell were washed twice with FBS staining buffer and resuspended in appropriate volumes of FBS staining buffer for fluorescent-activated cell sorting (FACS) analysis. Expression of these surface receptors was determined using an iCyt Synergy sorter system (Sony) in the UK Flow Cytometry Core Facility. Microglia, macrophages, and neutrophils were identified by CD11b^+^/CD45^lo^/GR1^lo/neg^, CD11b^+^/CD45^hi^/GR1^lo/neg^, and CD11b^+^/CD45^hi^/GR1^hi^ expressions, respectively [5, 6]. CD206 expression levels were used to determine M2-polarization states. For each antibody, gating was determined based upon appropriate negative isotype-stained controls. Flow data were analyzed using FlowJo software (Tree Star). Cell numbers for each animal were estimated from cell percentages and hemocytometer counts. All investigators involved in the flow/FACS analyses have been certified for flow research methods and applications through the completion of the Annual Course in Cytometry sponsored by the Cytometry Education Association and Verity Software House.

### Gene expression from FACS-sorted cells

All FACS-sorted macrophages (CD11b^+^/CD45^lo/hi^/GR1^lo/neg^), which consisted of both microglia- and monocyte-macrophages, were collected in FBS staining buffer (BD:554656), and 0.75 ml TRIzol LS reagent (Life Technologies) was added per 0.25 ml of suspension. Total RNA was isolated based on the manufacturer’s protocol, with an additional phase separation using BCP, precipitation with isopropanol (Sigma-Aldrich, St. Louis, MO), and wash of the isolated RNA in 70 % ethanol. Then, 1 μg RNA was reverse-transcribed using the high-capacity complementary (cDNA) reverse transcription kit (Life Technologies). Real-time PCR amplification was performed on the mixture of 100 ng cDNA sample, Taqman Universal PCR Master Mix, and Taqman Probes (Life Technologies) using the Applied Biosystems Step One Plus Real-Time PCR System. Probes included Arg1 (Mm00475988), CD206 (Mm00485148), and CD86 (Mm00444543). Expression of genes was normalized to 18S mRNA for each sample, and reported values were calculated as 2^-ΔΔCT^ relative to a sham reference sample.

### Behavioral analysis

All experimental animals were assessed using the Basso Mouse Scale (BMS) to score hindlimb function as previously described [23]. Mice were tested in an open field for 4 min before surgery and at 1, 3, 7, 14, 21, and 28 days post injury (dpi). Each hindlimb was scored separately based on movement (e.g., ankle placement and stepping), coordination, and trunk stability, and averaging both hindlimb scores generated a single score for each animal. A score of 0 indicated complete paralysis and a score of 9 indicated normal locomotion. Assessment of hindlimb function was also carried out using the gridwalk test [24]. The gridwalk utilizes a horizontal ladder with stainless steel rungs 4 mm in diameter spaced 1.2 cm apart. All experimental mice were trained before injury. On 27 dpi, only mice that could support their own body weight were tested on the apparatus. Animals were videotaped and evaluated on 30 continuous rungs on the center of the ladder. Frame-by-frame video analysis was used to track the total number of hindlimb steps/footfalls.

### Tissue processing and immunohistochemistry

Mice were anesthetized and then transcardially perfused with cold PBS (0.1 M, pH 7.4), followed by perfusion with cold 4 % paraformaldehyde (PFA). Dissected spinal cords (1 cm) were post-fixed for another 2 h in 4 % PFA and subsequently rinsed and stored in cold phosphate buffer (0.2 M, pH 7.4) overnight at 4 °C. On the following day, tissues were cryoprotected in 30 % sucrose for 3 days at 4 °C, followed by rapidly freezing and blocking in optimal cutting temperature (OCT) compound (Sakura Finetek USA, Inc.) on dry ice. Tissue was systematically randomized into blocks with equal group distribution to ensure uniformity of staining across groups, and blocked tissue was stored at −80 °C before sectioning. Tissue blocks were cut in serial coronal sections (10 μm) and mounted onto Colorfrost plus slides (Fisher #12-550-17). Spinal cord sections were stained for glial fibrillary acidic protein (GFAP) or neurofilament (NF) to measure tissue sparing. Slides were incubated with chicken anti-GFAP (1:200; Aves GFAP) or chicken anti-NF (1:1500, Aves NFH 0211) primary, followed by biotinylated goat anti-chicken (1:1000; Aves B-1005) then Alexa Fluor 488 (1:1000; Invitrogen S32354) secondary antibodies. Slides were coverslipped with Immu-Mount (Thermo Scientific, Waltham, MA). GFAP or NF fluorescent images were taken using a C2+ laser scanning confocal microscope (Nikon Instruments Inc., Melville, NY). To quantify spared tissue area, the regions of dense GFAP- or NF-positive staining were outlined and measured using the MetaMorph analysis program (Molecular Devices, Sunnyvale, CA).

### Cell culture

Bone marrow-derived macrophages (BMDMs) were extracted from the femur and tibia of female C57BL/6 mice at 8–10 weeks of age as previously reported [25, 26] and were plated at 0.8 ~ 1 × 10^6^ cells/ml in differentiation media: Dulbecco’s modified Eagle’s medium (DMEM) supplemented with 1 % penicillin/streptomycin, 1 % HEPES, 0.001 % β-mercaptoethanol, 10 % FBS, and 20 % supernatant from sL929 cells (a generous gift from Phillip Popovich, The Ohio State University). Supernatant collected from sL929 cells contains macrophage colony-stimulating factor, which helps to promote bone marrow cells’ differentiation into macrophages [27]. The BMDMs were allowed to differentiate for 7 days in culture, and cells were then replated on day 7 at a density of 1 × 10^6^ cells/ml in 12-well plates in differentiation media without L929 supernatant. On day 8, cells were stimulated to be M1 using LPS (50 ng/ml; Invivogen tlrl-eblps) plus IFNγ (20 ng/ml; eBioscience 14-8311-63) diluted in N2A growth medium. AZM (10 or 30 μM; Sigma PHR1088) or vehicle (DMSO; MP Biomedical 190186) was added at the time of stimulation. Unstimulated BMDMs were used as control. Six hours after incubation, the supernatant of the stimulated macrophages (macrophage-conditioned media (MCM)) was collected, filtered, then applied to cultured Neuro-2a cells or tested for IL-10 and IL-12p40 levels using standard ELISA kits (Thermo Scientific, Rockford, IL).

Mouse neuroblastoma cell lines (aka Neuro-2a or N2A, a gift from Chris Richards , University of Kentucky) were maintained in N2A growth medium containing 45 % DMEM, 45 % OPTI-MEM reduced-serum medium, 10 % fetal bovine serum (FBS), and 1 % penicillin/streptomycin. N2A were plated at a density of 1 × 10^5^ cells/ml in 48-well tissue culture plates and allowed to proliferate for 48 h. The neurotoxicity of MCM was evaluated as reported previously [7] using a MTT-based cell growth determination kit according to the manufacturer’s instructions (Sigma-aldrich). Briefly, on the day of testing, N2A growth media was replaced by fresh MCM, and the N2A cells were incubated in MCM for 24 h then thiazolyl blue tetrazolium bromide (MTT (5 mg/ml), 20 μl per well) was added to each well and the cells further incubated for 2 h. The tetrazolium ring of MTT can be cleaved by mitochondrial dehydrogenases of viable cells, yielding purple formazan crystals, which were then dissolved in acidified isopropanol solvent. The resulting purple solution was spectrophotometrically measured at 570 nm Epoch microplate reader (BioTek instruments, Inc., Winooski, VT) using 690 nm as a background absorbance. All measurements were done in triplicates, and at least three independent experiments were carried out.

### Statistical analysis

Investigators blinded to experimental conditions performed all data acquisition and analysis. Statistical analyses were completed using GraphPad Prism 6.0 (GraphPad Software). Data were analyzed using one- or two-way ANOVA followed by Holm-Sidak’s test for multiple comparisons. F-values are reported for repeated measures. Chi square and independent sample *t* tests were used when appropriate. Results were considered statistically significant at *p* ≤ 0.05. All data are presented as mean ± SEM unless otherwise noted. Figures were prepared using Adobe Photoshop CS6 (Adobe Systems) and Prism 6.0.

## Results

### AZM alters the macrophage response to SCI

**Fig. 1:**
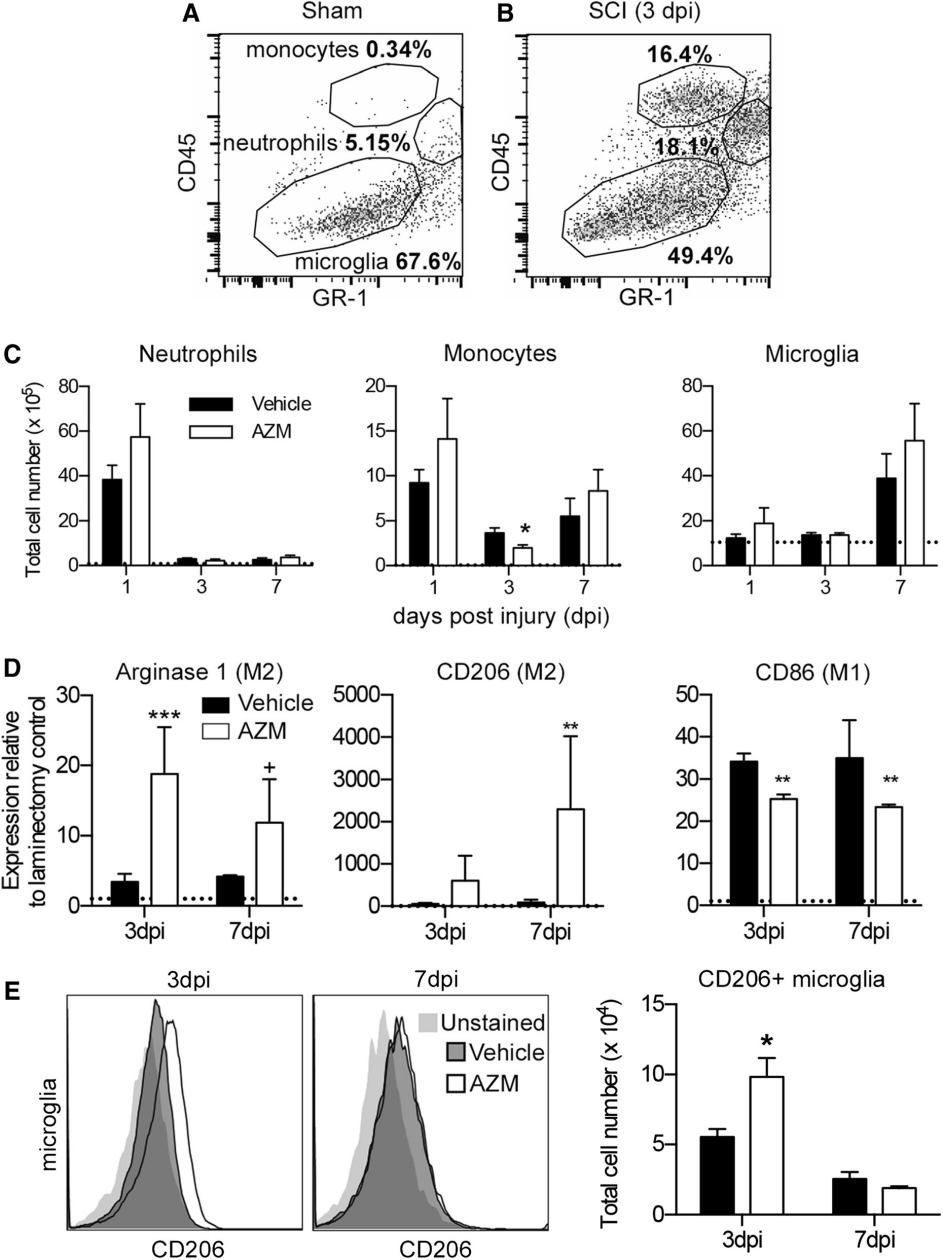
AZM treatment increases alternative macrophage activation in mouse SCI. Animals were subjected to a moderate-severe T9 SCI and treated with 160 mg/kg AZM or vehicle using a combined pre- (3 days) and post- (up to 7 days) SCI daily treatment regimen. **a**, **b** Scatter plots of CD11b/CD45+ cells isolated from spinal cord homogenates after sham or contusion SCI. Cells were collected from 10 mm of spinal cord tissue centered on the lesion epicenter. The specificity of the gates is evident as neutrophils and monocytes enter the spinal cord only after SCI (**b**). **c** Quantification of neutrophils (CD11b^+^/CD45^hi^/GR1^hi^), monocyte-derived macrophages (CD11b^+^/CD45^hi^/GR1^lo/neg^), and microglia (CD11b^+^/CD45^lo^/GR1^lo/neg^) (*gates indicated in* ***b***) following vehicle or AZM treatment (*1 dpi sham values indicated with dotted line*). AZM significantly attenuated monocyte recruitment to the spinal cord at 3 dpi (*p* = 0.03). **d** rtPCR of mRNA expression in FACS-sorted macrophages (combined monocyte and microglia populations identified in **b**) reveal significant increases in gene expression for the M2 markers arginase 1 and CD206 in AZM vs. vehicle controls (*values relative to sham expression levels indicated with dotted line*). The M1 marker, CD86, was significantly decreased with AZM treatment. **e** Representative plots for microglia-derived macrophages (CD11b^+^/CD45^lo^/GR1^lo/neg^) showing labeling with CD206 primary antibody and unstained controls. There is a significant increase in the number of CD206+ microglia-derived macrophages with AZM treatment. ^+^ *p* < 0.06, *p < 0.05, **p < 0.01, ***p < 0.001; *n* = 3–5

To determine the effects of AZM treatment on the inflammatory cell response to SCI, mice were treated daily with AZM or vehicle beginning 3 days prior to 75-kdyn T9 contusion SCI with continued daily administration (oral gavage) up to 7 days post injury (dpi). There were no significant differences between treatment groups in spinal cord displacement at the time of SCI (*p* > 0.35 for any given time point between groups and *p* = 0.50 for overall group effect). This is an important control to ensure all animals received comparable SCI regardless of pre-injury treatment. At 1, 3, or 7 dpi, cells were isolated from the injured spinal cord for phenotypic analysis (Fig. 1). Cells were labeled with antibodies specific for neutrophils (Gr-1) and macrophages (CD11b/CD45) then the relative numbers of neutrophils (CD11b^+^/CD45^hi^/GR1^hi^) and monocyte- (CD11b^+^/CD45^hi^/GR1^lo/neg^) and microglia- (CD11b^+^/CD45^lo^/GR1^lo/neg^) derived macrophages were quantified. As indicated in Fig. 1a, few monocyte-derived macrophages or neutrophils were detectable 1 day after sham SCI (also see dotted line in Fig 1c). Figures 1b, c show that the number of CD11b^+^/CD45^hi^/GR1^hi^ neutrophils in the injured spinal cord peaked at 1 dpi then decreased over time, CD11b^+^/CD45^hi^/GR1^lo/neg^ monocyte-derived macrophage numbers remained elevated over time, and CD11b^+^/CD45^lo^/GR1^lo/neg^ microglia increased from 1 to 7 dpi. In addition, AZM significantly attenuated the monocyte response to SCI at 3 dpi (Fig 1c, *p* < 0.05).

Next, we determined the phenotype of SCI macrophages using FACS to purify CD11b^+^/CD45^+^/GR-1^−^ macrophages from the spinal cord homogenates (both monocyte and microglia populations in Fig. 1b were pooled). Messenger RNA (mRNA) was isolated from the sorted, purified macrophages, and the relative gene expression of M2, anti-inflammatory (arginase, CD206), and M1, pro-inflammatory (CD86), macrophage markers was determined. Figure 1d shows that the expression of markers indicative of an anti-inflammatory phenotype, arginase and CD206, increased with SCI and AZM treatment significantly augmented expression of both arginase (F1,12 = 23.13, *p* = 0.0004) and CD206 (F1,12 = 11.86, *p* = 0.005) (Fig. 1d). Expression of the pro-inflammatory marker, CD86, increased with SCI and expression was attenuated by AZM treatment (F1,12 = 25.91, *p* = 0.003). There were no significant differences in macrophage phenotype between AZM- or vehicle-treated animals at 28 dpi (*p* > 0.8 for CD86 and arginase expression; Additional file 1: Figure S1).

Next, we examined if AZM was differentially increasing M2 marker expression on different macrophage populations. We used a CD206 antibody to determine the M2 profiles of monocyte- (CD11b^+^/CD45^hi^/GR1^lo/neg^) and microglia- (CD11b^+^/CD45^lo^/GR1^lo/neg^) derived macrophages isolated from the injured spinal cord at 3 and 7 days post-injury. Similar to what has been reported previously [6], microglia-derived macrophage expression of this M2 marker decreased over time (Fig. 1e). There was a significant time × drug interaction (F1,11 = 5.83, *p* = 0.03) with AZM significantly increasing the number of CD206+ microglia-derived macrophages relative to vehicle controls at 3 dpi (Fig. 1e, *p* = 0.01). This effect was not significant at 7 dpi (Fig. 1e, *p* > 0.6). In accordance with what has been previously reported for monocyte-derived macrophages [6], we observed similar levels of CD206 expression at both time points. Specifically, the numbers of CD206+ monocyte-derived macrophages were similar at 3 and 7 dpi, and there were no significant differences between AZM and vehicle at either time point (time × drug interaction (F1,11 = 0.28, *p* = 0.6)): 3 dpi, mean ± SEM: 1914 ± 156 (AZM) and 1988 ± 255 (veh); 7 dpi, 1945 ± 207 (AZM) and 1800 ± 155 (veh).

Collectively, these results indicate that AZM treatment alters the inflammatory response to SCI and decreases pro-inflammatory macrophage gene expression while potentiating anti-inflammatory macrophage gene expression. Further, AZM may potentiate microglia- vs. monocyte-derived M2 macrophage activation in response to SCI.

### AZM treatment improves locomotor recovery and decreases tissue pathology associated with SCI

**Fig. 2:**
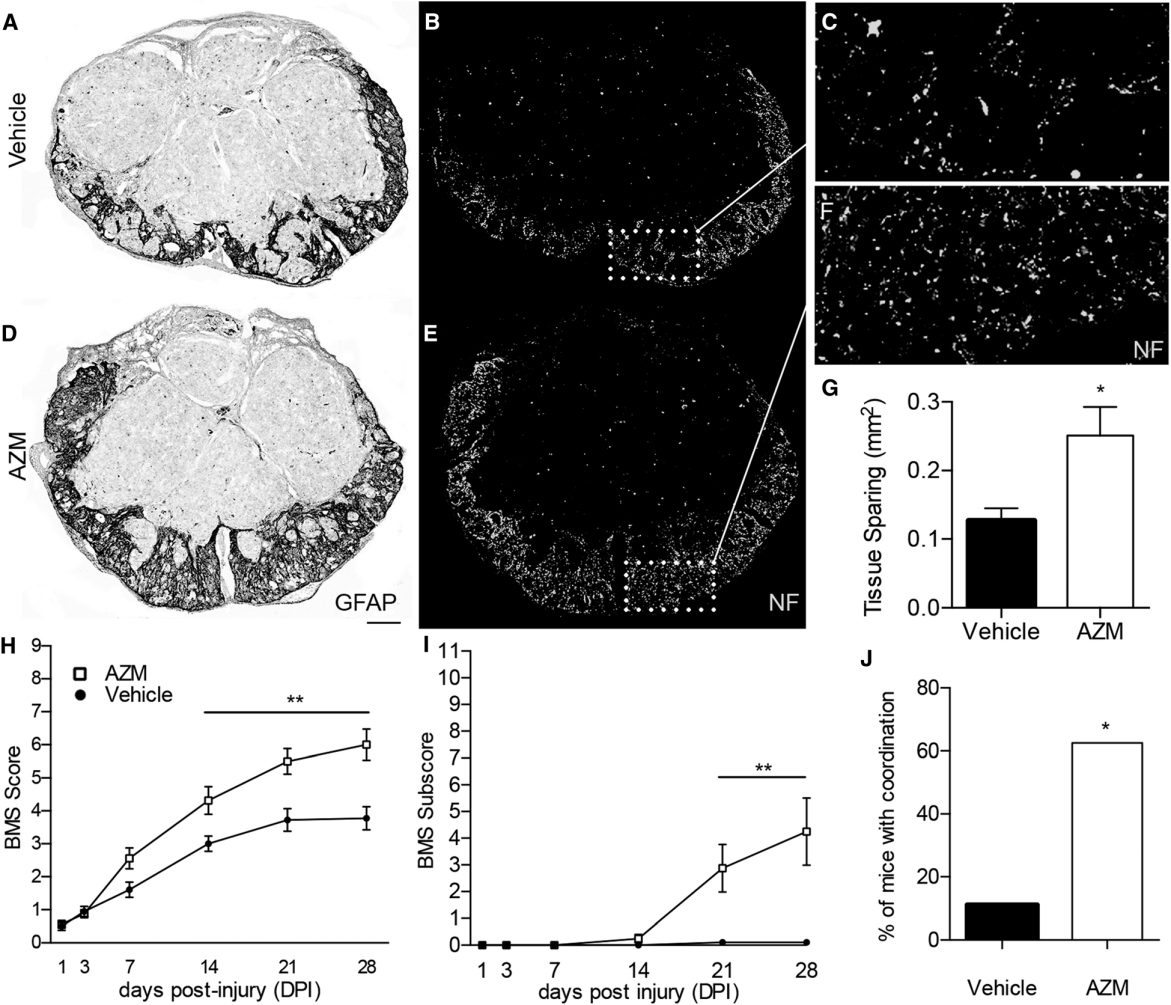
AZM treatment improves tissue sparing and recovery in SCI mice. Tissue sections representative of the mean values for vehicle- (**a**–**c**) and AZM- (**d**–**f**) treated animals at 28 dpi. **a**, **d** Inverted fluorescent images of GFAP-stained tissue sections of the lesion epicenter. **b**, **e** Adjacent sections stained with neurofilament (NF) indicate that areas of GFAP immunoreactivity correspond to areas of spared axons. **c**, **f** Higher powered confocal images of the rim of spared tissue reveal increased axon preservation with AZM treatment. **g** Quantification of tissue sparing at the lesion epicenter based upon GFAP immunoreactivity (*p = 0.01). **h**–**i** Functional recovery was assessed over a 28-day period using the Basso Mouse Scale (BMS). Mice treated with AZM recovered consistent stepping (BMS ~ 5) and coordination (BMS subscore ~4); vehicle-treated animals had some limb movement (BMS ~ 3) without coordination (BMS subscore = 0). **j** A significantly higher proportion of mice treated with AZM regained coordination of fore and hindlimbs compared to vehicle treated. *p < 0.05 vs. vehicle, **p < 0.01 vs. vehicle for each individual time point according to post hoc comparisons in response to the significant main treatment effect (*p* = 0.007). *n* = 8–10. Scale bar = 100 μm for **a**, **b**, **d**, **e** and 416 μm for **c**, **f**

Alterations in macrophage phenotypes are associated with differences in functional recovery and tissue sparing following SCI [6]. Because AZM treatment increased indices of anti-inflammatory macrophage activation, we assessed the ability of AZM treatment to improve SCI recovery. Figure 2a–g shows that AZM treatment resulted in significantly more spared tissue at 28 dpi. Specifically, the rim of spared tissue, defined using positive GFAP immunoreactivity, was roughly twofold greater following AZM (mean ± SEM = 0.25 ± 0.04 mm^2^) vs. vehicle treatment (0.13 ± 0.02 mm^2^) (*p* = 0.01). The area of neurofilament-positive spared axons was also significantly improved with AZM (0.21 ± 0.05 mm^2^) vs. vehicle treatment (0.098 ± 0.02 mm^2^) (*p* = 0.04).

Small differences in the amount of preserved tissue at the lesion epicenter after SCI correlate with significant differences in locomotor recovery [28]. To examine functional recovery in response to AZM treatment, locomotor function was assessed using the Basso Mouse Scale (BMS) [23]. As shown in Fig. 2h, following SCI, both treatment groups had significant functional deficits that improved over time (F5,75 = 179.7, *p* < 0.0001). There was significant overall treatment effect (F1,15 = 9.961, *p* = 0.007) and significant time × treatment interaction (F5,75 = 10.75, *p* < 0.001) meaning that AZM treatment significantly improved both the rate of recovery and overall level of residual deficits associated with SCI (Fig. 2h). Specifically, significant improvements were detected for AZM-treated mice compared to vehicle-treated by 14 dpi. At 28 dpi, AZM-treated animals had recovered consistent stepping (BMS ~ 5) while vehicle-treated animals had some limb movement without weight-supported stepping (BMS ~ 3). These improvements were also reflected in the BMS subscore, a supplement to the BMS scale that is sensitive to treatment-specific recovery progressions that may fall outside the normal pattern of recovery [23]. Specifically, there was a main effect of treatment (F1,15 = 10.93, *p* = 0.005) and time × treatment interaction (F5,75 = 11.76, *p* < 0.0001) that manifested at 28 dpi as fore-hindlimb coordination in the AZM group (BMS subscore ~4) with no fore-hindlimb coordination in the vehicle-treated group (BMS subscore ~0) (Fig. 2i).

Coordination is an important aspect of SCI recovery; therefore, we used a variety of motor tasks to determine if AZM treatment facilitated recovery of coordinated and proprioceptive function. First, a BMS score of 5 can indicate some level of coordination between fore- and hindlimb movements [23]. Figure 2 shows that all but one animal in the AZM-treated group achieved this score while only one animal in the vehicle group achieved coordination by 28 dpi (*p* = 0.002) (Fig. 2j).

**Fig. 3:**
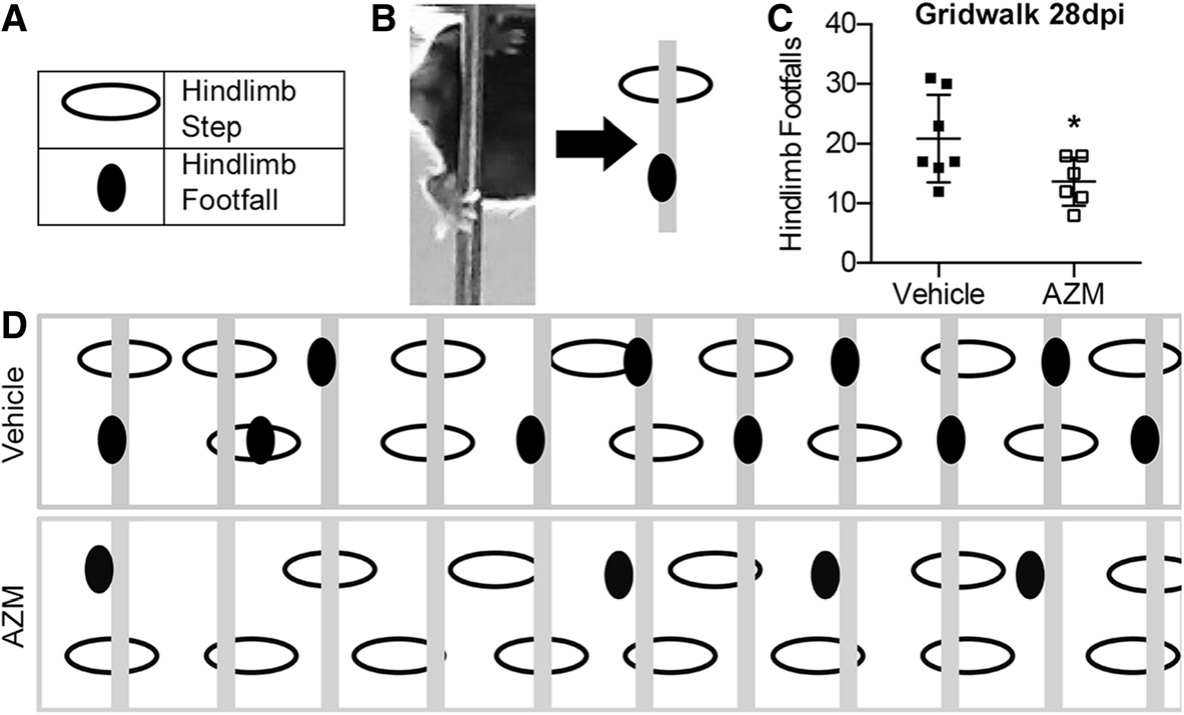
AZM treatment improves coordinated proprioceptive locomotor function in SCI mice. At 28 dpi, animals were tested on the gridwalk for proprioceptive coordination. **a**–**b** Frame-by-frame video analysis was used to track hindlimb steps/footfalls (*graphically represented as ovals*). **c** AZM-treated animals had significantly less footfalls than vehicle (*p* = 0.05; scored over 30 rungs). **d** Graphical representation of the footfall/stepping patterns over 11 rungs for one animal from each group representative of the mean performance of each group (*n* = 6–7)

Next, we assessed animals on the gridwalk task at 27 dpi. The gridwalk is a horizontal ladder task that measures sensory-motor coordination [29]. Two animals from the AZM group and three animals from the vehicle group did not achieve a sufficient level of recovery for gridwalk testing. Figure 3 shows that of animals able to perform the task, those treated with AZM had ~40 % fewer footfalls compared to vehicle-treated (*p* = 0.05). Overall, AZM treatment improved the locomotor function and coordination of mice recovering for contusion SCI.

### AZM alters the neurotoxicity of pro-inflammatory macrophages

Because we previously observed that pro-inflammatory macrophages are neurotoxic and likely potentiate secondary injury processes while anti-inflammatory macrophages do not cause cell death and drive repair processes [3], we next sought to determine if these neuroprotective effects could be mediated through AZM altering the toxic potential of pro-inflammatory macrophages.

**Fig. 4:**
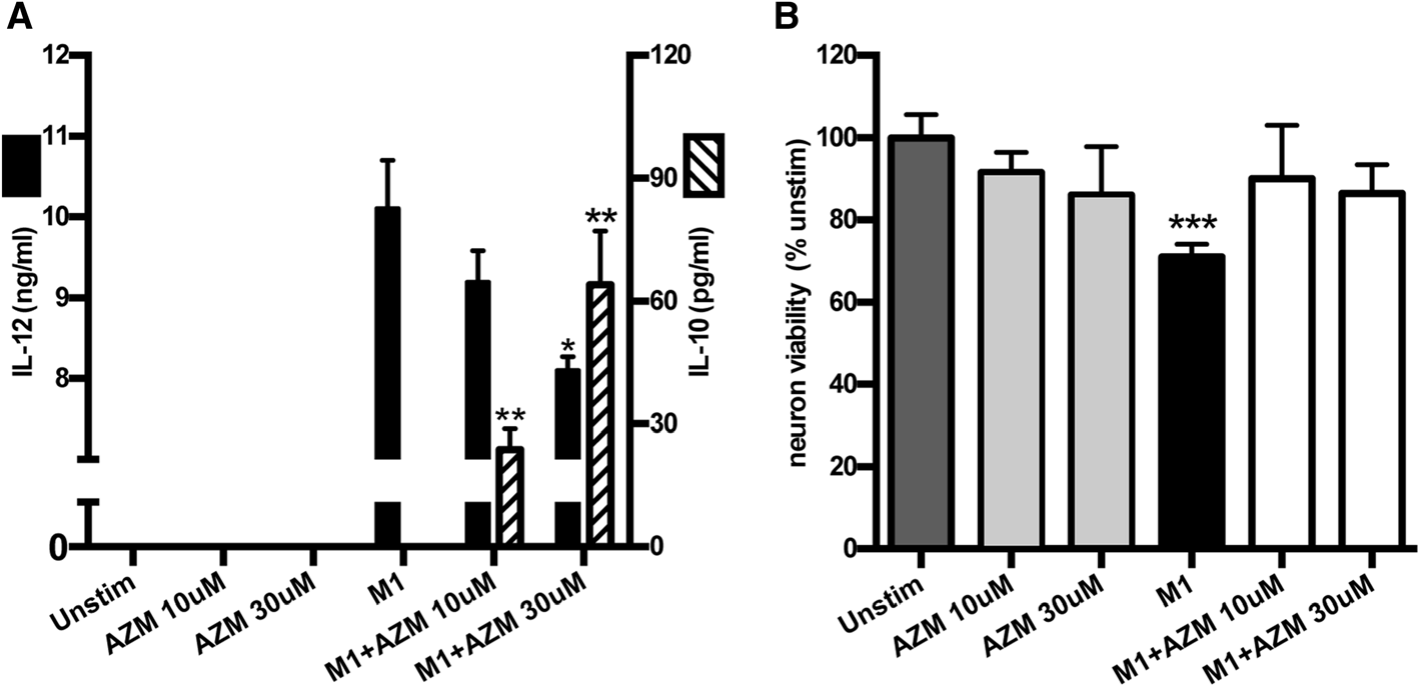
AZM treatment alters the phenotype and toxic potential of pro-inflammatory, M1 macrophages in vitro. Bone marrow-derived macrophages (BMDMs) were isolated from adult mice and stimulated to be M1 using LPS + INFγ in the presence or absence of AZM. Control cells were unstimulated or stimulated with AZM alone. **a** A high ratio of IL-12:IL-10 is a defining feature of M1 macrophages. AZM significantly decreased M1 production of the pro-inflammatory cytokine IL-12 and increased production of the anti-inflammatory cytokine IL-10. AZM alone had no affect on cytokine production. **b** MCM from M1 macrophages stimulated with AZM was not toxic to neurons. AZM alone did not significantly affect BMDM neurotoxicity (*p* > 0.2 for either AZM dose vs. *Unstim*). Data are representative of three independent replications. *p < 0.05, **p < 0.01 vs. M1. ***p < 0.001 vs. *unstim* and *p* < 0.05 vs. M1 + AZM groups. Mean ± SD

The effects of stimulating bone marrow-derived macrophages (BMDMs) in vitro are predictive of spinal cord macrophage responses in vivo [25, 26, 30]. Therefore, we examined the phenotype and neurotoxic potential of pro-inflammatory BMDMs treated with AZM in vitro. We used LPS + INFγ stimulation to model the pro-inflammatory, M1, macrophages that are activated in SCI [3, 6]. Figure 4a shows that AZM drives increased production of IL-10, an anti-inflammatory cytokine, while reducing pro-inflammatory IL-12 release from M1 macrophages. In the absence of the pro-inflammatory stimuli, AZM had no effect on unstimulated BMDMs (Fig. 4a). An increased ratio of IL-10:IL-12 is a defining feature of anti-inflammatory macrophages; therefore, we conclude from Fig. 4a that AZM drives pro-inflammatory macrophages toward an anti-inflammatory phenotype. As shown in Fig. 4b, this shift was associated with a significant decrease in the neurotoxicity of pro-inflammatory macrophages. Specifically, BMDMs were stimulated to be M1 in the presence or absence of AZM. The supernatants from these cells, deemed macrophage-conditioned media (MCM), were collected and used to treat neurons. The MCM from M1 cells resulted in significant reduction (~30 %, *p* = 0.001) in neuron viability relative to MCM from untreated cells. This toxicity was significantly attenuated with AZM treatment, as there was no significant difference in viability among neuron groups exposed to MCM from untreated vs. M1 + AZM-treated BMDMs (*p* > 0.15; Fig. 4b) or untreated vs. AZM-treated in the absence of M1 stimuli (*p* > 0.20; Fig. 4b). The results presented in Fig. 4 are representative of three independent experiments. Collectively, these results demonstrate that AZM can act directly on macrophages to alter pro-inflammatory activation and reduce macrophage-mediated neurotoxicity.

## Discussion

There is growing evidence that altering the phenotype of macrophages responding to SCI can improve recovery. Despite this, few safe pharmacological approaches have been identified that can manipulate SCI macrophages. Here, we show that in a mouse model, treatment with the macrolide antibiotic, AZM, results in increased macrophage expression of anti-inflammatory genes and facilitates significant improvements in SCI locomotor recovery and tissue sparing. Macrophages, purified from the injured spinal cord of mice treated with AZM, had increased expression of CD206 and arginase, indicators of an anti-inflammatory phenotype, with decreased expression of the pro-inflammatory marker CD86 (Fig. 1). AZM treatment significantly improved locomotor function compared to vehicle control, specifically in indices of locomotor coordination (Figs. 2 and 3). The improvements in locomotor recovery were associated with significant increases in tissue sparing (Fig. 2), presumably due to AZM reducing the neurotoxic potential of SCI-activated macrophages. Indeed, in vitro, AZM drove pro-inflammatory macrophages toward an anti-inflammatory phenotype with reduced neurotoxic properties (Fig. 4). Collectively, these data highlight the potential for an immunomodulatory, pharmacological therapy to be an effective treatment for SCI and identify AZM as a promising candidate for further translational development.

These key findings are consistent with observations of AZM-mediated changes in macrophage phenotype in models of lung infection, skin inflammation, and sepsis [17, 19, 31]; however, the results reported here are the first to document that AZM can have a similar effect after traumatic CNS injury. While pro-inflammatory macrophage activation is reduced with AZM treatment in acute conjunctiva [32], to the best of our knowledge, the current results are the first to demonstrate that AZM, or any other macrolide antibiotic, alters macrophage phenotype in response to spinal cord injury and reduces macrophage neurotoxicity. This is significant as neuroinflammation, and specifically pro-inflammatory macrophage activation, is a common feature of most neuropathological conditions including Alzheimer’s disease, stroke, aging, ALS, and traumatic brain injury [33–36]. The ability of AZM to be widely distributed in brain tissue following oral administration [37] makes it an intriguing candidate for manipulating macrophages in a variety of nervous system pathologies.

There is extensive data regarding the safety and dosing of AZM. Specifically, AZM is one of the antibiotics of choice for treating pneumonia in SCI individuals and is routinely administered at 10–45 mg/kg/day to treat infections in humans including community-acquired pneumonia, otitis media, and sinusitis [12, 13, 38]. Accounting for allometric scaling, the dose of 160 mg/kg used in the current study is high but still clinically relevant, especially considering that higher AZM doses should be tolerated if necessary for neuroprotection due to the drug’s large therapeutic window and limited toxicity profile. Additionally, the recent results of the “COPD: influence of macrolides on exacerbation frequency in patients” (COLUMBUS) clinical trial report that AZM can be administered chronically (for 12 months), albeit at lower doses, with maintained immunomodulatory effects and no increased adverse effects [39]. Ongoing work in our lab is examining the effect of lower doses of AZM on SCI inflammation and recovery.

One major limitation of the current work regarding the effectiveness of AZM for SCI treatment is that we utilize a combined pre- and post-SCI dosing strategy. We used this approach, based upon a previous dosing strategy we found to be effective for reducing inflammatory damage associated with acute lung infection [17], to test the proof-of-concept that AZM can effectively alter inflammation in response to CNS perturbations. Indeed, our findings provide evidence that initiating treatment prior to CNS inflammation is effective. This approach may be beneficial for altering inflammation in chronic neurodegenerative disease, e.g., aging and Alzheimer’s. This is especially relevant in light of the effectiveness of chronic AZM administration reported in the COLUMBUS study [39] and our previous observations that AZM treatment produces similar immunomodulary changes in both rodents and humans [17, 40]. We have made preliminary observations that AZM is effective when administration begins after SCI (unpublished data, manuscript in preparation). In addition, in the current study, AZM had no effect on macrophage phenotype in the absence of an inflammatory stimulus in vitro (Fig. 4). This is consistent with our previous observations, and that of others, that AZM does not polarize unstimulated macrophages [41]. Collectively, we therefore postulate that AZM treatment prior to SCI may not be required to facilitate improvements; however, additional studies are required to determine the therapeutic potential and required dosing for effective post-injury AZM treatment of SCI.

One observation in this study was that AZM treatment decreased monocyte-derived macrophages in the injured spinal cord. This is consistent with observations of reduced inflammation and macrophage accumulation with AZM treatment in models of acute conjunctiva, lung infection, and skin inflammation [17, 19, 32]. In addition, decreasing macrophage accumulation at the site of SCI is neuroprotective and facilitates recovery [42]. However, the mechanisms underlying the decreased macrophage recruitment we observed remain to be elucidated, as do the effects of AZM on monocyte- vs. microglia-derived macrophages.

Evidence that altering macrophage phenotypes or reducing M1 macrophage activation in SCI can be therapeutic comes from recent publications demonstrating that decreasing M1 macrophages in transgenic models of SCI leads to improved recovery, decreasing M2 macrophages or increasing M1 macrophages impairs SCI recovery, and increasing M2 macrophages using viral or transplantation approaches correlates with improvements in recovery [5, 6, 9, 43–45]. Our data demonstrate that the underlying mechanism mediating improvements in SCI recovery with AZM treatment may be due to its ability to reduce the neurotoxic potential and pro-inflammatory activation state of SCI macrophages. We have previously observed an M1 to M2 macrophage shift with AZM treatment in vitro [14]. The concept that AZM can shift macrophage phenotype is further supported by independent publications noting decreased IL-12, IL-6, IL-1β, TNF-α, and other pro-inflammatory mediators when macrophages are stimulated in the presence of AZM [15, 21, 46–49]. Interestingly, these publications suggest that M1, but not M2, macrophage activation is affected by AZM. It is also possible, given the combined pre-and post-SCI dosing strategy, that AZM prevented M1 polarization in the current study rather than altering the M1 to M2 phenotype. This concept is support by the observation that AZM inhibits signaling cascades specific to pro-inflammatory stimuli [50]. The specific mechanism responsible for the immunomodulatory potential of AZM and other macrolides are not well understood. Nonetheless, these collective observations indicate that AZM may selectively attenuate pro-inflammatory macrophage activation.

The neuroprotective effects we report with AZM are similar to the effects reported for SCI treatment with the antibiotic minocycline [51]. Identifying neuroprotective, non-minocycline antibiotics has important implications for SCI therapeutic translation and treatment. There is an inherent risk of developing antimicrobial resistance with any antibiotic use, especially in SCI individuals who often undergo repeated antibiotic treatments to fight recurrent infections [13, 52]. In addition, higher adverse reaction rates are associated with minocycline vs. other antibiotic treatments [53]. Therefore, the identification of neuroprotective and antibiotic alternatives to minocycline increases the probability that these drugs can be used as neuroprotective strategies for treating SCI.

It is also important to identify the immunomodulatory mechanism of actions in order to develop more potent neuroprotectants. Due to structure differences between tetracycline (minocycline) and macrolide (azithromycin) antibiotics, it is difficult to imagine that both antibiotics are working through similar mechanisms of action. Attenuated pro-inflammatory microglial activation, however, has been reported with minocycline treatment in vitro [54]. Interestingly, similar to AZM, minocycline selectively affects M1 but not M2 macrophages. Inhibition of the NF-κB pathway in pro-inflammatory macrophages has been observed for both antibiotics [41, 54]. In addition, this is a somewhat common feature of other macrolide antibiotics [50]. It is worth investigating the common structural elements of macrolides, and potentially other antibiotics, that mediate these immunomodulatory effects. Identifying the necessary structural components of macrolides that effect macrophage biology would provide insight into pro-inflammatory macrophage activation while facilitating development of more potent therapies that do not have the potential for causing antimicrobial resistance. There is evidence that modified macrolides retain their immunomodulatory properties [55–58], and we are currently exploring the ability to use these or other novel macrolide compounds to facilitate CNS repair.

We recently reviewed the potential positive impact of M2 macrophages on SCI wound repair [2]. Moving forward, it is important to determine mechanistically whether the benefits observed in SCI through AZM treatment are due primarily to the reduction of inflammatory factors and M1 macrophage activity or if there is a specific beneficial function of the M2 macrophage that AZM potentiates. A more regulatory battery of cytokine production, including IL-10 and TFGβ, is typical with M2 cells [59, 60]. In addition, M2 macrophages can actively inhibit inflammatory processes through arginase-1 up-regulation, which competes with inducible nitric oxide synthase (iNOS) for l-arginine [61, 62]. These studies demonstrate the ability of M2 macrophages to suppress iNOS production, reduce inflammatory cytokine/chemokine secretion, and control neutrophil infiltration [63]. However, in our own studies of cystic fibrosis patients, we observed that chronic AZM treatment significantly lowered inflammatory gene expression including iNOS and TNF-α, but did not significantly increase M2-associated gene expression [40]. The future goal of our studies is to characterize the complicated role of anti-inflammatory macrophages over the entire post-injury time course.

## Conclusions

The findings presented here demonstrate that AZM can alter the macrophage response to SCI and facilitate improve tissue sparing and functional recovery. We also demonstrate that AZM directly alters the phenotype and neurotoxic potential of pro-inflammatory macrophages in vitro. AZM is safe at high doses, can be administered chronically without adverse effects, and accumulates in brain tissue following oral administration. These features make AZM an ideal drug for treating neuroinflammatory conditions. Additional studies are needed to determine the effectiveness of post-injury administration, optimize the dose and therapeutic window for SCI, and examine the mechanisms through which AZM alters inflammation. Our data in mice, nonetheless, indicate that AZM is a promising therapy for treating SCI.

## Additional file

Additional file 1: Figure S1. AZM treatment does not lead to long-term changes in macrophage activation or phenotype after SCI. Representative tissue sections of vehicle- (**A**, **D**, **F**) and AZM- (**B**, **E**, **H**) treated animals at 28 dpi. **A**, **B** Fluorescent images of Tomato Lectin (TomL)-stained sections (1:1000, Cat# L0651, Sigma-Aldrich) reveal no significant difference in the density of macrophage activation between groups as quantified in **C**. (Note for **C**, **F**, and **I**, proportional area was quantified and normalized to vehicle). **D**, **E** Fluorescent images of the same sections as **A**, **B** stained for CD86 (1:100, Cat# 553689, BD Pharmingen, Franklin) show no differences between groups as quantified in **F. G**, **H** Fluorescent images of the adjacent sections to **A**,**B**/**D**,**E** stained for arginase 1 (1:200, Cat # sc-18354, Santa Cruz Biotech) show no difference between groups as quantified in **I**. Scale bar = 100 μm, *n* = 8–10.
