# Supplementary figures and images for: Azithromycin drives alternative macrophage activation and improves recovery and tissue sparing in contusion spinal cord injury
Source: J Neuroinflammation. 2015 Nov 24;12:218. doi: 10.1186/s12974-015-0440-3 (PMC4657208; doi:10.1186/s12974-015-0440-3)

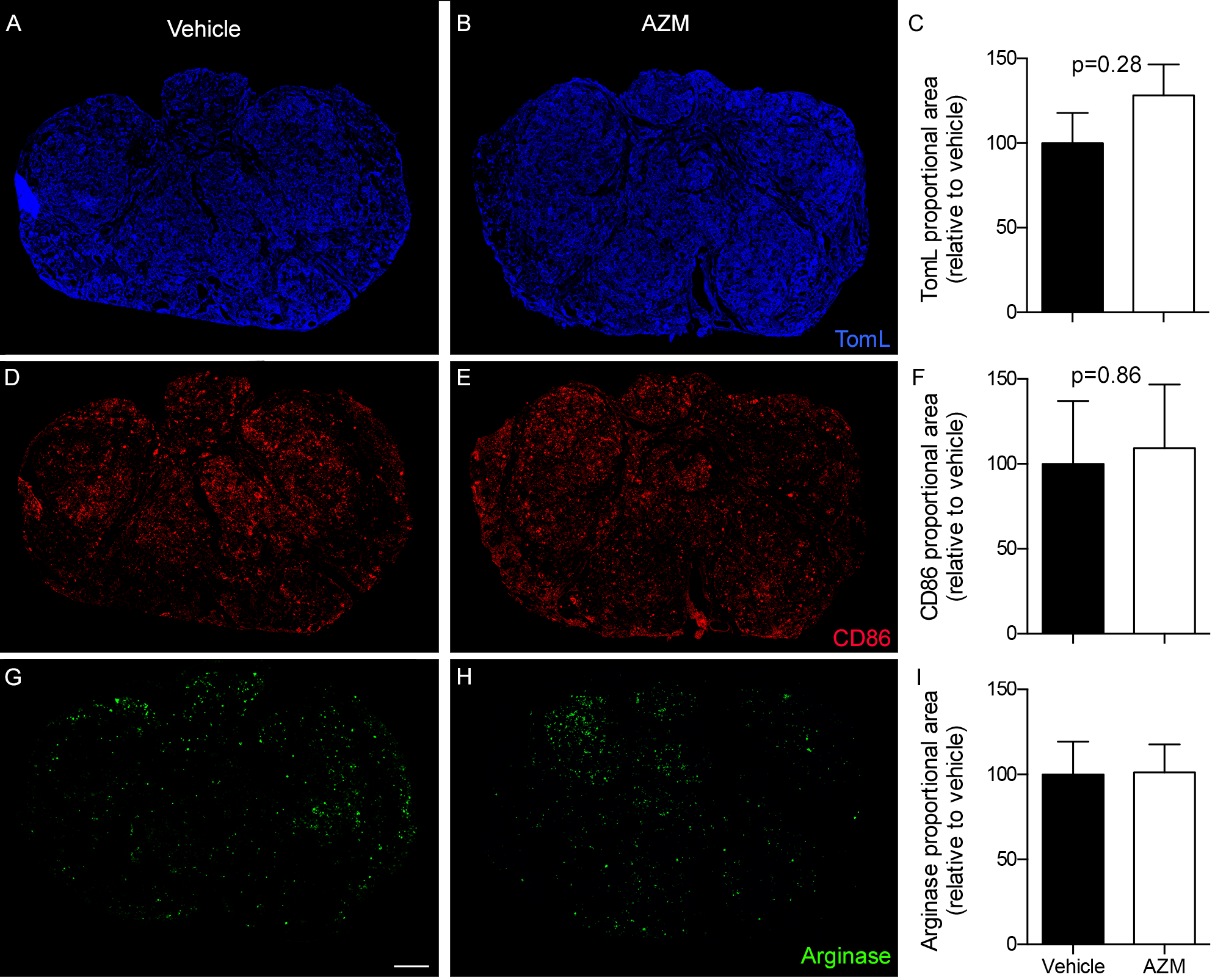

Supplement: Additional file 1: Figure S1. — AZM treatment does not lead to long-term changes in macrophage activation or phenotype after SCI. Representative tissue sections of vehicle- (A, D, F) and AZM- (B, E, H) treated animals at 28 dpi. A, B Fluorescent images of Tomato Lectin (TomL)-stained sections (1:1000, Cat# L0651, Sigma-Aldrich) reveal no significant difference in the density of macrophage activation between groups as quantified in C. (Note for C, F, and I, proportional area was quantified and normalized to vehicle). D, E Fluorescent images of the same sections as A, B stained for CD86 (1:100, Cat# 553689, BD Pharmingen, Franklin) show no differences between groups as quantified in F. G, H Fluorescent images of the adjacent sections to A,B/D,E stained for arginase 1 (1:200, Cat # sc-18354, Santa Cruz Biotech) show no difference between groups as quantified in I. Scale bar = 100 μm, n = 8–10. [file 12974_2015_440_MOESM1_ESM.tif]
